# Healthy Diet Promotion through Evidence-Informed Policies

**DOI:** 10.3390/nu15112514

**Published:** 2023-05-29

**Authors:** Ruopeng An, Chen Chen

**Affiliations:** Brown School, Washington University, St. Louis, MO 63130, USA; c.chen2@wustl.edu

The Special Issue entitled “The Impact of Policy and Food Environment on Food Purchase and Dietary Behavior” comprises 13 articles that collectively provide valuable insights into the complex interplay between policy, food environment, and individual food purchase and consumption. This editorial briefly summarizes common themes, highlights key trends and findings emerging from the articles, and discusses their implications for future research, policy, and practice.

Several articles within this Special Issue investigate the impact of policy interventions on food purchasing patterns and dietary behavior. Hawkins et al., Ferris et al., and Chen and Yang explore the effects of the Supplemental Nutrition Assistance Program (SNAP), the Community Eligibility Provision (CEP), Breakfast after the Bell (BATB), and the National Nutrition Program 2017–2030. These studies demonstrate the potential for policy interventions to promote healthier food choices and improve nutrition outcomes, particularly among vulnerable populations [1,2,3]. Additionally, Barker et al. highlight the influence of national nutrition policies on consuming sugar-sweetened beverages, emphasizing the need for comprehensive, multi-sectoral approaches to address global nutrition challenges [4].

The food environment also plays a crucial role in shaping dietary behavior and health outcomes. McCarthy et al., McKerchar et al., Reyneke et al., Racine et al., and Salvo et al. explore the relationship between the food environment, purchasing patterns, and dietary behavior. These studies underscore the importance of considering geographic and economic factors when designing interventions to improve access to healthy foods [5,6,7,8,9]. In particular, Salvo et al. highlight the potential impact of policies that jointly increase geographic and economic access to fruit and vegetables among low-income, predominantly Latino urban residents [9].

In this Special Issue, several articles focus on the role of food outlets in shaping food choices and diet quality. For example, Vinyard et al. and Bergman et al. examine the relationship between different types of food outlets and the Healthy Eating Index-2015 scores, as well as menu engineering and dietary behavior among young adults. These findings suggest that strategies to improve diet quality and promote healthier food choices should consider the role of food outlets and their potential influence on consumer decision-making [10,11]. In addition, Mijares et al. and Machida highlight the significance of food waste among consumers, and non-market fruit and vegetables sold outside grocery stores could contribute to consumers’ daily intake and reduce food waste [12,13].

Several key themes and trends emerge from the articles included in this Special Issue. First, policy interventions can potentially shape food purchasing patterns and dietary behavior, particularly among vulnerable populations. This highlights the importance of developing and implementing evidence-based policies that effectively address the complex determinants of dietary behavior and health outcomes. Second, the food environment significantly influences individual food choices, with geographic and economic factors shaping access to healthy foods. Interventions to improve the food environment should consider these factors to promote equitable access to nutritious options. Third, the role of food outlets in shaping dietary behavior cannot be underestimated. Efforts to improve diet quality and promote healthier food choices should consider the influence of food outlets, including menu design and the availability of healthy options. Finally, a comprehensive, multi-sectoral approach is necessary to address the complex interplay between policy, food environment, and individual dietary behavior. Collaboration among policymakers, researchers, and practitioners is essential to develop and implement effective strategies that improve nutritional outcomes and promote healthier, more sustainable food systems.

In sum, this Special Issue provides a wealth of insights into the complex interactions between policy, food environment, and dietary behavior. The findings underscore the need for a comprehensive, multi-sectoral approach to address global nutrition challenges, focusing on developing and implementing evidence-based policies and interventions that consider the diverse factors shaping food choices and health outcomes. As we move forward, it is crucial for future research to build on the findings from this Special Issue and explore the potential synergies and trade-offs between different policy interventions and food environment strategies. This will help ensure that interventions are designed to maximize health benefits while minimizing unintended consequences. Moreover, engaging with diverse stakeholders, including policymakers, food industry actors, and community organizations, will be essential for developing and implementing effective strategies that are grounded in the needs and realities of different populations and contexts.
